# Analysis of factors influencing cervical lymph node metastasis of papillary thyroid carcinoma at each lateral level

**DOI:** 10.1186/s12893-022-01678-w

**Published:** 2022-06-15

**Authors:** Wen-qing Liu, Jing-yi Yang, Xiao-hui Wang, Wei Cai, Fei Li

**Affiliations:** 1 Xuan Wu Hospital of The Capital Medical University, Beijing, China; 2 Capital Medical University, Beijing, China; 3 General Surgery Department, Xuanwu Hospital, No. 45 Changchun Street, Xicheng District, 100053 Beijing, China

**Keywords:** Papillary thyroid carcinoma, Neck dissection, Lateral cervical metastases

## Abstract

**Objective:**

To analyze the clinicopathological characteristics of patients with papillary thyroid carcinoma (PTC) and its influence on the distribution of lymph node metastasis at each lateral level of the neck to guide precise treatment of the lateral area.

**Methods:**

The clinicopathological data of patients with PTC initially diagnosed and treated at our hospital from February 2014 to September 2021 were collected; the metastatic status of each lateral level was recorded, and correlations were analyzed.

**Results:**

A total of 203 patients were enrolled in this study. There were 67 males and 136 females, with an average age of 41.1 years. In the lateral cervical area, lymph node metastasis was found at level IIa in 81 patients (39.9%); level III, 171 patients (84.2%); level IV, 122 patients (60%); and level Vb, 18 patients (8.9%). Correlation analysis showed that age (r = 0.198, P < 0.01) and sex (r = 0.196, P < 0.01) were weakly correlated with the number of positive lymph nodes in the central region. The tumor size (r = 0.164, P < 0.05) was weakly correlated with lymph node metastasis at level IV. The presence of multiple tumor foci was weakly correlated with lymph node metastasis at level IIa (r = 0.163, P < 0.05) and Vb (r = 0.143, P < 0.05). The tumor location (r = − 0.168, P < 0.05) was weakly correlated with lymph node metastasis at level III. The number of positive lymph nodes in the central region (r = 0.189, P < 0.01) was weakly correlated with lymph node metastasis at level IV. Binary logistic regression analysis showed that the risk of metastasis of multifocal tumors was higher than that of unifocal tumors by 1.958 times at level IIa (P = 0.021, OR = 1.958) and 2.929 times at level Vb (P = 0.049, OR = 2.929). The higher the tumor was located, the higher the risk of metastasis at level III (P = 0.014, OR = 0.563). Every additional positive lymph node in the central region increased the risk of metastasis at level IV by 1.126 times (P = 0.009, OR = 1.126).

**Conclusions:**

For patients with pathological evidence of lateral metastasis, standard dissection of level IIa through Vb is recommended; selective dissection requires careful consideration. Patients with multifocal tumors have a high risk of metastasis at levels IIa and Vb, which requires special attention during the operation.

According to the latest data released by GLOBOCAN in 2020, the incidence of thyroid cancer ranks ninth among all tumors, with 10.1 cases/10 million women and 3.1 cases/10 million men, which is significantly higher than that 10 years ago [1]. As society is paying increasing attention to this disease, ultrasound examination of the thyroid has gradually become more common. More cases of early-stage thyroid cancer are being screened and diagnosed, and papillary thyroid carcinoma (PTC) is the most common type. Approximately 20% of all PTC cases are accompanied by lateral lymph node (LLN) metastases at the initial diagnosis [2, 3], accounting for 18.6% (203/1091) of all cases in the Thyroid and Breast Center of our hospital. The range of LLN dissection has evolved many times over the years; the latest American Thyroid Association (ATA) guidelines recommend that patients with LLN metastases should undergo level IIa, III, IV, and Vb dissection [4]. Recently, the value of selective dissection or super-selective dissection has been discussed, since dissection at level IIa or Vb may cause spinal accessory nerve injury, but few studies have explored the predictive factors for the distribution of metastatic lymph nodes at each lateral level. To identify these predictive factors and to provide further evidence to guide the accurate treatment of the lateral cervical region, this study analyzed the records of all patients who had LLN metastasis at the initial diagnosis and underwent standard level IIa-Vb LLN dissection from 2014 to 2021 at our center.

## Methods

We collected data from PTC patients who underwent ipsilateral therapeutic neck dissection of LLNs, including concomitant total thyroidectomy, between February 2014 and September 2021. A total of 203 patients diagnosed and treated at our center between February 2014 and September 2021 were included in this study. There were 67 males and 136 females, with an average age of 41.1 years. Patient demographic data are shown in Table 1. Institutional Review Board approval was obtained for this study. The inclusion criteria were as follows: (1) confirmation of the primary lesion as PTC by postoperative pathology according to the pathological criteria of the 2015 ATA Management Guidelines for Adult Patients with Thyroid Nodules and Differentiated Thyroid Cancer; (2) treatment with level IIa-Vb LLN dissection; and (3) complete available clinical and pathological records. The exclusion criteria were as follows: (1) other pathological types of thyroid malignancies; (2) distant metastasis; (3) previous history of surgery for malignant thyroid tumor; and (4) presence of other malignant tumors.

**Table 1:** Demographic profile and clinicopathological factors of the PTC patients

Characteristics	Value
Age (years)	41.12 ± 12.9
Sex (n)	
Female	136
Male	67
Size (cm)	2.0 ± 1.3
Tumor (n)	
Unifocal	103
Multifocal	100
Pathological subtype (n)	
Classical	184
Follicular	11
Eosinophilic	1
Clear cell	1
High cell	6
Side (n)	
Left	96
Right	107
Location (n)	
Upper	108
Middle	53
Lower	42
Capsular invasion (n)	
Yes	188
No	15
Positive CLN (n)	4.8 ± 3.7
Level IIa (n)	
Positive	81
Negative	122
Level III (n)	
Positive	171
Negative	32
Level IV (n)	
Positive	122
Negative	81
Level Vb (n)	
Positive	18
Negative	185

All patients in this study were evaluated by ultrasonography and enhanced computed tomography (CT) of the neck. LLN metastases were proven by fine-needle aspiration (FNA) or intraoperative frozen pathology. All operations were completed at the Thyroid and Breast Center of our hospital. The surgical procedures performed included or total thyroidectomy, ipsilateral central lymph node (CLN) dissection, and therapeutic lateral cervical lymph node dissection (levels IIa through Vb). Central region dissection included the prelaryngeal and pretracheal lymph nodes and paratracheal lymph nodes. The upper border of the dissection area was the hyoid bone, and the lower border was the innominate artery. The range of LLN dissection was in accordance with the recommendations of the ATA 2012 consensus and included levels IIa, III, IV, and Vb.

The Kolmogorov–Smirnov test showed that all data followed a nonnormal distribution. Spearman correlation analysis was used to explore the correlation between several clinicopathological factors and the metastatic status of each level (IIa through Vb). Binary logistic regression was used to analyze the relationship between risk factors and LLN metastasis at each level. P value < 0.05 was considered to indicate statistical significance. SPSS 23.0 (SPSS, Inc., Chicago, IL, USA) was used to analyze the data.

## Results

In the lateral cervical area, lymph node metastasis was found at level IIa in 81 patients (39.9%); level III, 171 patients (84.2%); level IV, 122 patients (60%); and level Vb, 18 patients (8.9%).

Correlation analysis showed in Table 2 that age (r = 0.198, *P* < 0.01) and sex (r = 0.196, *P* < 0.01) were weakly correlated with the number of positive lymph nodes in the central region. The tumor size (r = 0.164, *P* < 0.05) was weakly correlated with lymph node metastasis at level IV. The presence of multiple tumor foci was weakly correlated with lymph node metastasis at level IIa (r = 0.163, *P* < 0.05) and Vb (r = 0.143, *P* < 0.05). The tumor location (r = − 0.168, *P* < 0.05) was weakly correlated with lymph node metastasis at level III. The number of positive lymph nodes in the central region (r = 0.189, *P* < 0.01) was weakly correlated with lymph node metastasis at level IV. Binary logistic regression analysis showed in Tables 3 and 4 that the risk of metastasis of multifocal tumors was higher than that of unifocal tumors by 1.958 times at level IIa (*P* = 0.021, OR = 1.958, 95% CI: 1.107–3.463) and 2.929 times at level Vb (*P* = 0.049, OR = 2.929, 95% CI: 1.004–8.547). Table 5 showed that metastasis at level IIIwas 0.563 times more likely for tumors located lower in the lobe than tumors located higher in the lobe (*P* = 0.014, OR = 0.563, 95% CI: 0.356–0.889). Table 6 showed that every additional positive lymph node in the central region increased the risk of metastasis at level IV by 1.126 times (*P* = 0.009, OR = 1.126, 95% CI: 1.030–1.231).

**Table 2:** Correlation analysis of clinicopathological factors and metastasis at each level

		Age	Sex	Size	Multifocal or unifocal	Side	Location	Capsular invasion	CLN
Level IIa	r	0.018	− 0.016	0.010	0.163^*^	0.067	− 0.063	0.076	0.044
	*P*	0.804	0.824	0.893	0.020	0.345	0.373	0.279	0.529
Level III	r	0.105	0.045	0.080	0.021	− 0.004	− 0.168^*^	0.085	0.082
	*P*	0.134	0.525	0.259	0.770	0.959	0.017	0.231	0.248
Level IV	r	0.024	0.058	0.164^*^	0.119	− 0.026	0.087	0.116	0.189^**^
	*P*	0.736	0.407	0.020	0.092	0.710	0.217	0.100	0.007
Level Vb	r	0.002	0.039	0.092	0.143^*^	0.018	− 0.022	0.022	0.069
	*P*	0.982	0.580	0.190	0.041	0.801	0.752	0.757	0.329

− 0.500 < r < 0.500 or : weakly correlations

*P = 0.05 **P = 0.01

**Table 3:** Binary logistic regression analysis of clinicopathological factors and lymph node distribution at level IIa

Variables	*P*	Exp(B)	95% CI Exp(B)	
			Lower	Upper
Multifocal or unifocal	0.021	1.958	1.107	3.463
Constant	0.002	0.241		

**Table 4:** Binary logistic regression analysis of clinicopathological factors and lymph node distribution at level Vb

Variables	*P*	Exp (B)	95% CI Exp (B)	
			Lower	Upper
Multifocal or unifocal	0.049	2.929	1.004	8.547
Constant	0.000	0.017		

**Table 5:** Binary logistic regression analysis of clinicopathological factors and lymph node distribution at level III

Variables	*P*	Exp (B)	95% CI Exp (B)	
			Lower	Upper
Location	0.014	0.563	0.356	0.889
Constant	0.000	15.050		

**Table 6:** Binary logistic regression analysis of clinicopathological factors and lymph node distribution at level IV

Variables	*P*	Exp (B)	95% CI Exp (B)	
			Lower	Upper
Size	0.057	1.276	0.992	1.640
CLN	0.009	1.126	1.030	1.231
Constant	0.071	0.539		

## Discussion

Thyroid glands have plentiful lymphatic vessels; thus, cervical lymph node metastasis is regularly observed and specific to PTC. Generally, PTC first metastasizes from the primary tumor site to the pretracheal, paratracheal and upper mediastinal lymph nodes, then to the ipsilateral LLNs, and finally to the contralateral region [5]. Skip metastasis occurs when tumor cells bypass the CLNs, and metastasis first occurs in the LLNs. This phenomenon has been reported in several studies, affecting approximately 21.8–23.5% of patients [6, 7]. In this study, 13 of 203 patients with lateral metastasis had skip metastasis. Lateral metastasis is suggestive of a poor prognosis. In this study, the probability of lymph node metastasis in the lateral area was 39.9% at level IIa, 84.2% at level III, 60% at level IV and 8.9% at level Vb. This is consistent with the 53% probability of metastasis at level II, 71% at level III, 66% at level IV and 25% at level V reported in the literature [8].

 The ATA first published guidelines for the treatment of thyroid nodules and differentiated thyroid cancer in 1996 and updated these guidelines in 2006, 2009 and 2015. The range of central neck dissection was defined in the 2009 ATA guidelines [9] and has not been revised to date. The upper boundary is the hyoid bone, the bilateral boundary is the medial margin of the common carotid artery, and the lower boundary is the innominate artery, including lymph nodes at levels VI and VII. For the treatment of lymph nodes in the lateral cervical region, George Crile first proposed radical neck lymph node dissection in 1906, including lymph nodes at levels I-V and resection of the internal jugular vein, accessory nerve and sternocleidomastoid muscle. Because of the severe surgical trauma that can occur with this approach, along with many complications and poor postoperative quality of life, modified cervical lymph node dissection is now widely used. For differentiated PTC, the ATA recommended in 2012 that patients with LLN metastasis should undergo lymph node dissection at levels IIa, III, IV and Vb. Dissection of lymph nodes at level IIb or Va should be performed only if there is clear evidence of metastasis at these two levels [4]. The latest National Comprehensive Cancer Network (NCCN) guidelines [10] for the range of lateral neck lymph node dissection are consistent with the ATA guidelines. The patients enrolled in this study were diagnosed and treated from 2014 to the present, and the surgical procedures were performed in strict adherence with the standards outlined in the 2012 ATA guidelines.

In this study, the number of positive lymph nodes in the central region was a risk factor for lymph node metastasis at level IV. Previous studies have suggested that CLN metastasis can predict the possibility of overall lateral metastasis [11–13]; however, no studies have yet reported the predictive effect of CNL metastasis at each level of the lateral cervical region. The results of this study suggest that the number of positive CNLs has predictive value for level IV metastasis. For each additional positive lymph node in the central region, the risk of metastasis at level IV increased by 1.126 times. However, the number of positive lymph nodes in the central region could not be accurately confirmed by preoperative imaging [14, 15]. Moreover, all the patients included in this study had pathological evidence of LLN metastasis before lateral dissection. Level IV is a conventional dissection region. The frozen pathology results of the number of positive lymph nodes in the central region during surgery could not provide additional guidance for selection of the surgical strategy. For patients without pathological evidence of lateral metastasis before lateral dissection, if the number of suspected positive CLNs during the operation is high, and this suspicion is confirmed by frozen pathology, does this indicate a risk for metastasis at level IV? Should this guide the surgeon in evaluating the lateral region? To answer these questions, further confirmation by a prospective study is needed.

The lateral cervical lymph node dissection range has been modified several times. The reduction in the surgical scope has been brought on by the application of precision therapy according to evidence-based medicine. Prolonging the overall survival of patients, reducing postoperative complications and improving the quality of life of patients have always been the goals of surgery. Investigation into whether the LLN dissection scope can be further reduced, such as in selective level dissection or super-selective level dissection, is ongoing [16, 17]. Some researchers believe that super-selective lymph node dissection is feasible, especially in robot-assisted and endoscopic surgery [16, 18], while other researchers doubt the safety of this approach. Doctor Piccin [19] suggests that patients with preoperatively proven PTC LLN metastasis should undergo dissection including levels II to V and that the transoral robotic approach may not be the ideal surgical technique for neck dissection. In a study conducted by Doctor Zhao [16], the experimental group underwent selective dissection at levels III and IV, and the control group underwent standardized dissection at levels II through V. The rate of lymph node metastasis at level II in the control group was 33% (42/147). Although 1/3 of the patients in the experimental group were spared from level II dissection, the risk of recurrence could not be ruled out without close long-term follow-up. Our results revealed that the risk of metastasis of multifocal tumors was higher than that of unifocal tumors by 1.958 times at level IIa (*P* = 0.021, OR = 1.958) and 2.929 times at level Vb (*P* = 0.049, OR = 2.929). The risk of metastasis at levels IIa and Vb is significantly higher for multifocal tumors than unifocal tumors. In cases of multifocal tumors detected on preoperative imaging, more attention should be given to these two levels when performing LLN dissection. Can patients with a unifocal tumor be spared from level IIa and Vb dissection? The data in the present study showed that, in cases of unifocal tumors, 5 patients were positive for metastasis at level Vb; 33 patients, level IIa; and 2 patients, levels IIa and Vb. Among them, only 2 patients were pathologically diagnosed with level IIa or Vb metastases before the operation. Other diagnoses were made incidentally after the surgery. Therefore, with selective dissection, 16.7% (34/203) of patients with a unifocal tumor would have been at risk of residual metastatic lymph nodes at levels IIa and Vb. We suggest that for patients with unifocal tumors, if selective dissection is considered, careful evaluation by ultrasonography and enhanced CT of levels IIa and Vb is critical. If any suspicious lymph node is observed, FNA should be performed to guide the dissection range and avoid residual positive lymph nodes.

This study has the following limitations. First, the time span of this study was long; some surgeons used monopolar electrosurgical units, some used ultrasonic knives, and some used bipolar electrosurgical units. Bipolar electrosurgical units are more accurate anatomically and may allow the removal of more lymph nodes. Second, because the number of positive lymph nodes at each level varied widely across patients, the metastatic status of lymph nodes in each lateral region was recorded as negative or positive for research and analysis. If the absolute number of positive lymph nodes or the proportion of positive lymph nodes is recorded and analyzed, different results may be obtained [20], but further evidence is needed to demonstrate that a certain data recording method is more clinically objective. In addition, this study was retrospective in nature. Further prospective studies with higher evidence levels are needed in the future.

## Conclusions

 At present, for patients with PTC and pathological evidence of lateral cervical metastasis, standardized dissection from level IIa through level Vb is recommended according to the ATA guidelines. Especially for patients with multifocal tumors, the metastasis risk at levels IIa and Vb is high and requires special attention. More evidence of the safety of selective LLN dissection is needed to prove its feasibility.

## Data Availability

The datasets generated during and analyzed during the current study are not publicly available due to no IRB’s approval of data sharing but are available from the corresponding author on reasonable request.
